# Social and Cultural Challenges in Caring for Latinx Individuals With Kidney Failure in Urban Settings

**DOI:** 10.1001/jamanetworkopen.2021.25838

**Published:** 2021-09-17

**Authors:** Lilia Cervantes, Katherine Rizzolo, Alaina L. Carr, John F. Steiner, Michel Chonchol, Neil Powe, Daniel Cukor, Romana Hasnain-Wynia

**Affiliations:** 1 Division of Hospital Medicine, Denver Health, Denver, Colorado; 2 Office of Research, Denver Health, Denver, Colorado; 3 Division of General Internal Medicine, University of Colorado Anschutz Medical Campus, Aurora; 4 Department of Renal Diseases and Hypertension, University of Colorado Anschutz Medical Campus, Aurora; 5 Department of Psychology, University of Colorado, Denver; 6 Institute for Health Research, Kaiser Permanente Colorado, Aurora; 7 Department of Medicine, Priscilla Chan and Mark Zuckerberg San Francisco General Hospital, San Francisco, California; 8 Department of Medicine, University of California, San Francisco; 9 Rogosin Institute, New York, New York

## Abstract

**Importance:**

Latinx individuals with end-stage kidney disease (ESKD) constitute 19% of US patients receiving in-center scheduled hemodialysis. Compared with non-Latinx White patients, Latinx individuals often face poor economic, environmental, and living circumstances. The challenges for health care professionals engendered by these circumstances when Latinx individuals present with ESKD and possible solutions have not been well described.

**Objective:**

To examine the perceptions of interdisciplinary health care professionals who work in dialysis centers in urban settings with large racial and ethnic minority populations about how social challenges affect the care of Latinx patients with ESKD.

**Design, Setting, and Participants:**

This qualitative study administered semistructured interviews of interdisciplinary health care professionals at 4 urban dialysis centers in Denver, Colorado, from April 1 to June 30, 2019. Interviews were audio recorded, transcribed verbatim, and analyzed using thematic analysis.

**Main Outcomes and Measures:**

Themes and subthemes of barriers to care.

**Results:**

Thirty interdisciplinary dialysis center health care professionals (23 [77%] female; mean [SD] age, 42.0 [11.6] years) participated. Four themes were identified. The first 3 themes and their respective subthemes (in parentheses) describe challenges to kidney care: compromised quality of care attributable to communication and cultural barriers (language interpretation by telephone, in-person language interpretation, burden of ad hoc interpretation, low-quality health care, lack of language- and culturally concordant materials, and health literacy levels), difficulty with health care access (unreliable transportation, economic instability, and loss of insurance benefits), and concerns about patient psychosocial well-being (social isolation, hopelessness, stigma of illness, and balancing personal social challenges). The fourth theme describes solutions to improve care (culturally responsive care, patient empowerment and activation, supporting primary caregivers, and peer support with navigation of the health care system).

**Conclusions and Relevance:**

This study’s findings suggest that dialysis center policies are needed that require high-quality language interpretation and the availability of culturally concordant educational materials. Community-based interventions that improve patient activation and provide peer support as well as culturally responsive care may improve the care of Latinx patients with ESKD receiving in-center scheduled hemodialysis.

## Key Points

**Question:**

What are the perceptions of dialysis center health care professionals about the social challenges to care faced by Latinx individuals with kidney failure receiving hemodialysis?

**Findings:**

This qualitative study, through interviews with health care professionals, identified barriers to care of Latinx individuals with kidney failure, including communication issues attributable to low use of interpreters and lack of language- and culturally concordant materials, as well as difficulty with health care access. Those interviewed also identified facilitators to care that included providing culturally responsive care.

**Meaning:**

The results of this study suggest that policies that require high-quality language interpretation and the availability of culturally concordant educational materials are needed in the dialysis setting.

## Teaser

This qualitative study interviewed interdisciplinary health care professionals at urban dialysis centers to assess how social and cultural challenges affect the care of Latinx patients with kidney disease receiving thrice-weekly hemodialysis.

## Introduction

The Latinx community constitutes 19% of individuals with end-stage kidney disease (ESKD) receiving in-center scheduled hemodialysis thrice weekly in the US.^1^ Social challenges (ie, economic, environmental, and living conditions) are potential determinants of health that are associated with poor outcomes among members of racial and ethnic minority groups with kidney disease.^2,3,4,5,6,7,8,9,10,11,12,13,14^ Latinx (ie, Hispanic, preferred non–gender-based term for Latino/Latina) individuals often face additional challenges, such as language barriers, lack of culturally concordant educational materials, and discrimination.^15,16,17,18,19,20,21^ Among Latinx individuals with chronic kidney disease, an estimated 66% report limited English proficiency (LEP), but little is known about the availability of language interpreters.^22^ The Latinx ESKD community is heterogeneous, representing a wide variety of national origins and cultural groups; little is known about the availability of culturally concordant educational materials.^22,23,24^ Regarding immigration status, many Latinx US citizens with ESKD live in mixed immigration-status families, and many report fear of discrimination.^19,20,25,26^

One aspect of improving the quality of care and health outcomes for this population is to understand the perceptions of interdisciplinary health care professionals who work in dialysis centers in urban settings with large racial and ethnic minority populations and are at the forefront of providing care to Latinx individuals.^27,28^ Health care professionals are often inadequately equipped to address their patients’ social challenges but have insight into barriers and facilitators to kidney care. In this study, we describe the perceptions of health care professionals at inner-city dialysis centers about how social challenges affect care. This work could inform new local and national dialysis center interventions to improve care for Latinx individuals with ESKD.

## Methods

### Study Design, Participants, and Settings

In this qualitative study, we conducted interviews with interdisciplinary health care professionals who had provided at least 1 year of direct care at an inner-city dialysis center in Denver, Colorado. Participants were recruited by flyers, email, and word of mouth from 4 different dialysis organizations. Recruitment materials stated that the study would assess perspectives on social challenges to kidney care. Purposive sampling captured a diverse sample (ie, different demographic characteristics and disciplines). We initially accepted volunteers in the order in which they reached out to participate but then capped volunteers from certain clinical categories to ensure a balanced number of health care training backgrounds. Participants provided informed consent. All data were deidentified. The University of Colorado multi-institutional review boards approved this study. This study followed the Consolidated Criteria for Reporting Qualitative Research (COREQ) reporting guideline.^29^

### Data Collection

Face-to-face, qualitative interviews were conducted from April 1 to June 30, 2019 (L.C.). The interview guide (eTable in the Supplement) was based on a literature review of social challenges faced by Latinx patients with ESKD.^16,18,19,30^ Interviews were audio recorded and transcribed verbatim. Participants were recruited, and analysis of data was conducted until thematic saturation was reached.^31^

### Statistical Analysis

Interview transcripts were imported and analyzed using ATLAS.ti, version 8.0.27.0 (Scientific Software Development). Coding and analysis were conducted according to the principles of grounded theory and thematic analysis.^31,32^ Line-by-line coding was performed to inductively identify initial concepts. Similar concepts were grouped into initial themes and subthemes, and then conceptual links among themes were identified (L.C. and A.L.C.). Consensus on themes and subthemes was reached after review of the thematic analysis and discussion of the themes to ensure that the findings reflected the full range and depth of the data (L.C., A.L.C., J.F.S., and R.H.-W.).^31,33^

## Results

### Participants and Characteristics

Thirty health care professionals (23 [77%] female; mean [SD] age, 42 [11.6] years; mean [SD] time at an inner-city dialysis center, 9.8 [6.4] years) from 4 dialysis centers participated. Participants included physicians (n = 6), registered nurses (n = 8), hemodialysis technicians (n = 7), social workers (n = 4), registered dietitians (n = 3), a nurse practitioner (n = 1), and a physician assistant (n = 1) (Table 1). The mean (SD) interview duration was 48 (11) minutes.

**Table 1.** Demographic Characteristics of 30 Interdisciplinary Dialysis Center Health Care Professionals^a^

Characteristic	Finding
Age, mean (SD), y	42.0 (11.6)
Female sex	23 (77.0)
Male sex	7 (23.3)
Race and ethnicity	
Asian	2 (6.6)
Black	2 (6.6)
Latinx	9 (30.0)
Non-Latinx White	17 (56.6)
Bilingual	12 (40.0)
Time providing direct dialysis care, mean (SD), y	9.8 (6.4)
Discipline	
Clinical social worker	4 (12.3)
Hemodialysis technician	7 (23.3)
Registered dietitian	3 (10.0)
Physician assistant	1 (3.3)
Nurse practitioner	1 (3.3)
Registered nurse	8 (26.6)
Physician	6 (20.0)

^a^ Data are presented as number (percentage) of participants unless otherwise indicated.

We identified 4 themes. The first 3 themes describe social challenges to kidney care. The fourth theme describes solutions to mitigate barriers to kidney care. The respective subthemes are described below; supporting quotations are provided in Table 2.

**Table 2.** Themes and Subthemes With Illustrative Quotes From Health Care Professionals

Themes and subthemes	Illustrative quotes
**Compromised quality of care attributable to communication and cultural barriers **	
Language interpretation by telephone	“Most patients are not comfortable with it [telephone interpretation]. It really would be so much easier and more comfortable if you had someone that spoke their language in front of them. It would be nice if some of the clinicians could speak other languages.” (nurse) “Sometimes the phone interpreter [doesn’t] speak very loudly so it’s not ideal.” (registered dietitian)
In-person language interpretation	“[Our dialysis organization] prefers that we use the language line because it is less costly than an in-person language interpreter…sometimes they allow us to have an in-center interpreter but only once per month.” (social worker) “Some patients don’t respond well to the phone interpreter and in those cases, I’ll try to arrange an in-person interpreter. It’s amazing how much better they respond to the in-person interpreter than someone on the phone.” (registered dietitian)
Burden of ad hoc interpretation	“There’s nobody here to interpret. There are two of us that speak Spanish and I know that when we’re not here to help translate but they don’t use a phone interpreter. I don’t even know that the dialysis staff know what’s available, honestly. These patients don’t understand the importance of their renal therapy because there’s no one there to interpret. They have no idea what they’re being told. That’s why I try to be there to interpret for them.” (hemodialysis technician) “I’ve seen the dialysis technicians cry…they are told, ‘I need you to interpret for me right now’ and the technicians have to drop what they are doing.” (registered dietitian) “I have a good relationship with the Hispanic patients. I am there for them when they have questions and I’m there for them trying to make sure that they understand things correctly. For some reason, when they use the phone interpreter, they just nod their heads. I have to go and reiterate whatever was just said…When I’m on the floor running around doing my tech care, time gets tight.” (hemodialysis technician)
Low-quality health care	“I see limited English proficiency as a huge barrier. So many different languages. I can sometimes figure out what Spanish speakers are saying but instructing new patients on a diet that don’t understand English is a barrier.” (registered dietitian) “We definitely weren’t giving him the best care that he needed because we just couldn’t communicate or educate him like we needed to.” (registered dietitian) “They are not comfortable calling the transplant center because they are worried the information will be in English. They’re not going to call online because they don’t have a computer or they don’t know how to use it. We have good tools for them online but it’s a barrier. If they can’t access this information, they won’t ever have a transplant.” (hemodialysis technician)
Lack of language- and culturally concordant materials	“You’re sending them home with a handout in a totally different language and you’re expecting them to be adherent? How do you expect them to use this at home? I just wish that there was a way we could have all of these material in Spanish, for different cultures, so that everyone is getting information and not feeling they’re stuck or confused.” (registered nurse) “When I need informational diet documents in Spanish, I go to a dietitian national listserv. I can’t get it from my dialysis organization because it’s not available.” (registered dietitian) “Our dialysis organization puts out some dietary information in Spanish but only for an American diet with foods that Americans eat. I spend time Googling things like ‘what do Puerto Rican people eat?’…we need to find educational materials that are tailored.” (registered dietitian)
Health literacy levels	“[Low health literacy] prevents them from understanding what we’ve recommended…so, it’s not that they didn’t hear what was being said but it wasn’t explained in a way that made sense.” (physician)
**Difficulty with health care access **	
Unreliable transportation	“Everyone that’s poor has issues with transportation. It’s always been an issue. Our city is particularly horrible with transportation for these poor patients.” (registered nurse) “90% of the time, my patients are late because transportation was late or didn’t show up. I feel so bad.” (registered nurse) “Sometimes their treatment runs 5 minutes longer for whatever reason and they are left here in dialysis and transportation won’t wait. I think it’s just a few companies that are contracted through Medicaid and Medicare.” (physician assistant)
Economic instability	“And then I have patients who will cancel other appointments because they know that their Medicare is going to pay 80% and they're going to get billed for 20% or when they walk into the office they are going to get a higher co-pay. That was another conversation I had this morning with a patient. He was really upset. He's been paying these co-pays for other doctors’ appointments and he can't afford them.” (social worker)
Loss of insurance benefits	“If I don’t think that the patient can fill out paperwork at home and they don’t have family that can help them out either, then I sit down with them and help them fill out paperwork. I also have financial coordinators that can help them fill out other financial paperwork.” (social worker) “Most of my patients are on Medicaid and food stamps and many of them bring their paperwork so that I can help them fill it out. The paperwork isn’t in their language even after they request in their language or sometimes they don’t have the education and can’t read or understand the paperwork. I need to do this otherwise their Medicaid benefits and food stamps, just stop.” (social worker)
**Concerns about patient psychosocial well-being **	
Social isolation	This patient fell and I asked her, ‘Did you tell your daughter?’and she responded, ‘Oh, she’s busy.’ The patient says that she is home all day by herself.” (hemodialysis technician) “Patients don’t want to be a burden on friends or family. Many patients don’t have a good family and don’t live with family. A lot of them are isolated.” (social worker)
Hopelessness	“If they feel depressed, they don’t care about their health and they don’t come to treatment. There’s a patient that said, ‘I’m done, I don’t want to do dialysis no more. If I don’t get on the transplant list again, I’m just going to *put* myself on hospice.’” (hemodialysis technician) “A lot of young people don’t see any hope, there’s no future, and they feel like they are losers or worthless. I think it’s a lot of depression and the way it manifests is with nonadherence because they don’t have insight. They kind of know on one level but they don’t have the emotional maturity to take care of themselves because it just doesn’t matter.” (physician)
Stigma of illness	“The dietary restrictions that they always tell me about and especially culturally the foods that they want to eat, that they like to eat, they can't eat. That's going to affect how you feel and how you can participate in your family gatherings when you're not supposed to be eating certain things and everybody else is eating them.” (physician) “I've learned over time that the word *depression* scares the Hispanic limited English proficient community. Or they’re like 'Depressed? I’m not depressed.’ So I try to give other options, other words to describe depression.” (hemodialysis technician)
Balancing personal social challenges	“We have a young woman that has so much going on. She has 3 kids with an ex-husband and son in jail. As a mom, she puts her needs last and those of her kids first. So, she puts dialysis on the back burner because she has to put all of this other stuff first. I kind of understand why she’s not in dialysis and why it’s not a priority for her. She has so many issues.” (physician assistant) “They have basic needs like food, utilities, rent and that’s all really hard. It’s like when Jesus had a sermon on the mountains and he was asked, ‘Why are you feeding the people?’ and he responded, ‘You can’t educate people or teach people when they are hungry.’ Our patients are hungry for these basic needs. If they can’t get their basic needs met, I can’t talk to them about these higher-level needs that would improve their health. They are not going to be interested in talking to me about their goals or mental health issues because they are just trying to survive. They are worried about not having enough food or their utilities have been cut off, or they can’t get their medications, or don’t have transportation.” (social worker)
**Solutions to improve care **	
Culturally responsive care	“We have an immigrant that never received education. She doesn’t speak anything but her native language. I told this NP [nurse practitioner], ‘You’re talking to her in English and you’re giving her information in English. You’re talking down to her and then you’re calling her stupid? It’s not that she’s stupid. You’re not communicating with her well.’ So, these are things that we struggle with that take a lot of time to resolve to prevent harm because you have a provider that is getting upset with patients that don’t speak English instead of taking their time to realize that there are language, education, and cultural barriers.” (social worker) “The cultural context is important, you know? You might have a young woman that doesn’t want a fistula because she doesn’t want to be deformed. I can say, ‘I get it, it looks really ugly, right?’ I needed to empathize and then help her understand that having it would be better than a higher mortality.” (physician) “I’ve learned a lot that I can break down barriers a little by trying to speak Spanish. I know that I make mistakes but then I say, ‘Teach me.’ That hopefully might break down the barrier of authority, just seeing these white coats and stuff, I think it’s silly that we have to wear these.” (social worker)
Patient empowerment and activation	“And I think sometimes you lose focus. Just because this clinic, they get audited by the state, and I know that there’s a lot of things that we can work on to improve and make sure that this clinic can be better. And I just think that people just need to focus on, okay, we’ll do the policy but don’t lose track of the patients. Because the patients, these are human beings, they’re not just, you know, like things that come and go. Like these are people that actually want to.” (hemodialysis technician) “I say, ‘What motivates you to miss dialysis? What motivates you to come? What are your goals?’” (nurse practitioner)
Supporting primary caregivers	“I feel like to truly understand the need for renal therapy, they need medical knowledge, and if a family member is present, that is really helpful. We don’t know if they talk about their illness with their family. So, if spouse or child comes to dialysis, we try to have the conversation.” (physician assistant) “We try to help the family understand why dialysis is a good thing. We want to work towards getting family more involved. Sometimes, getting dialysis care can be a strain on the family. The family comes and drops them off and then picks them up. So, it’s good to make sure that the family is doing okay because we don’t want this to strain the relationship between the patient and the family. Caregivers can face a lot of stress and guilt.” (hemodialysis technician)
Peer support with navigation of the health care system	“Many of us can’t relate but someone who comes from their community would have an easier time connecting and could talk to them about kidney transplantation and maybe have more of an impact than the rest of us.” (physician)

### Compromised Quality of Care Attributable to Communication and Cultural Barriers

Health care professionals noted communication barriers, including difficulty with telephone interpretation, lack of in-person interpretation, burdening bilingual staff, and concerns about compromising quality of care.

#### Language Interpretation by Telephone 

Several health care professionals described difficulty developing personalized and trusting relationships with patients when using telephone interpretation. One health care professional noted that “patients were more candid” with a live interpreter. The health care professionals also described technical issues with telephone interpretation because initiating the calls was time-consuming and some patients who were hard of hearing had difficulty participating. One dialysis technician said, “Patients are more candid with an in-person interpreter compared to a phone interpreter. When we’re using the phone interpreter, patients might only complain of pain but maybe there are three things they want to talk about and would bring up but they don’t because it takes too long to pass the phone.”

#### In-person Language Interpretation

Face-to-face language interpretation was preferred but described as cost-prohibitive: “My dialysis organization would not be happy if I requested an in-person interpreter because it’s expensive.” When patients requested an in-person interpreter for an important conversation, such as dialysis withdrawal or end-of-life care, they needed to schedule this “weeks in advance…because we are told that it costs a lot of money.”

#### Burden of Ad Hoc Interpretation

Spanish-speaking staff, who often had technical roles, such as dialysis technicians, reported being asked to step into the role of interpreter; because of power dynamics, they felt compelled to interpret as an expansion of their duties despite balancing other clinical responsibilities: “It just gets to be too much…they say, ‘Call this patient.’ They even tell me to go through forms in Spanish. I’m not compensated.” Health care professionals described using dialysis technicians for language interpretation and justified this because there was no alternative.

#### Low-Quality Health Care

Health care professionals described how patients with LEP received suboptimal care: “There is no one to translate. If there’s no Spanish speaker…then the patient isn’t getting the medication changes they need, the appointments, the referrals. This impacts their care, treatment, and quality of life.” Health care professionals also described how LEP affected kidney transplantation: “We referred one of our Spanish-speaking patients for a transplant. He called the intake line but there was nobody who spoke Spanish…you can’t get a visit until you do the intake.”

#### Lack of Language- and Culturally Concordant Materials

Health care professionals noted a lack of language- and culture-concordant educational materials. Health care professionals described commonly used tools, such as the Kidney Dialysis Quality of Life (KDQOL) and Patient Health Questionnaire 2, as difficult to use because the questions were not translated in culturally concordant ways: “I hate giving the KDQOL survey because the questions don’t make sense to them. When I try to explain it to them, they look at me like I’m from Mars…but they want to comprehend.” Registered dietitians were frustrated by the lack of culturally tailored dietary materials.

#### Health Literacy Levels

Health care professionals described struggling to provide health information to the varying health literacy levels of patients as well as those with poor vision: “It’s important to find educational tools that make sense to the patient. There are patients that can’t read and sometimes they won’t tell you that they can’t read or that their vision is bad. It’s a sensitive issue for them.”

### Difficulty With Health Care Access

Health care professionals described patient struggles with self-care because of unreliable transportation, economic instability, and losing insurance benefits when they did not meet application eligibility deadlines.

#### Unreliable Transportation

Health care professionals noted that unreliable transportation contributed to missed and shortened hemodialysis sessions. Transportation companies were often late for patient pickups or forgot them altogether. In addition, transportation companies would not wait for patients to complete hemodialysis: “They either don’t come, or when they do come, and if the patient isn’t quite off the machine, they leave.”

#### Economic Instability

Some health care professionals stated that finances limited patient ability to adhere to medication regimens, follow restricted diets, and keep clinic appointments: “We’ve struggled with my patient’s blood pressure for 2 months now… she tells me, ‘I haven’t been able to pick up [the medications] because they’re too expensive.’” Health care professionals also described frustration trying to navigate patient deductibles: “At the beginning of the year, patients haven’t met their Medicare deductible and they say, ‘This medication costs too much.’”

#### Loss of Insurance Benefits

Health care professionals described frustration with Medicaid when patients’ benefits were dropped. Social workers described how the loss of insurance benefits disproportionately affected people who could not fill out yearly paperwork: “Some patients can’t read their mail because they are blind, illiterate, or English is not their primary language…and they don’t know that they have to return the renewal Medicaid or Medicare paperwork.”

### Concerns About Patient Psychosocial Well-being

Health care professionals noted how patients undergoing dialysis expressed social isolation and hopelessness and struggled with the cultural stigma of having kidney disease. Health care professionals also described how some Latinx patients struggled to balance multiple social challenges.

#### Social Isolation

Several health care professionals explained the importance of family support on patient well-being: “I dig into their home situation…I have found that there’s a direct correlation between how well our patients do in dialysis and how well they are supported by people outside of dialysis.” Health care professionals also described how patients often explained that they did not want to be a burden to their family and thus did not ask for help with transportation or medications.

#### Hopelessness

Health care professionals observed that patients who seemed hopeless and/or depressed were not concerned about adherence to medications and hemodialysis. One health care professional noted that when “patients feel hopeless, they question everything: ‘what’s the point?’…some feel like they will always be on dialysis, so why bother taking pills?’”

#### Stigma of Illness

Health care professionals described many patients struggling with stigma. For example, patients shared not wanting to be singled out when needing to eat different foods and not wanting to be questioned when taking phosphorus binders during meals: “I had a patient that worked, and he didn't want to take his phosphorus binders in front of his construction-work friends. He didn't want them to know.” Health care professionals also shared how their ability to diagnose and intervene in mental health issues was limited by patient stigma: “It’s also a taboo for my Mexican patients to say they’re depressed...I wonder if we are missing opportunities to diagnose depression and so perhaps we should do this in a different way.”

#### Balancing Personal Social Challenges

Health care professionals noted that patients missed hemodialysis sessions because of myriad social challenges that they prioritized over dialysis. “Patients may be trying to get food on the table or are facing severe socioeconomic struggles…I think it’s hard, in that setting, to elicit a shared understanding of the importance of an intervention like dialysis.”

### Solutions to Improve Care

Health care professionals described ways to improve care for their Latinx patients: providing care that is culturally responsive, patient activation as a means to improve patient self-care, and providing support for caregivers and peer support for patients.

#### Culturally Responsive Care

Many health care professionals described how awareness and understanding of the influence of culture on attitudes, beliefs, and experiences fostered cross-cultural communication and improved patient care. “You have to know how different cultures understand and interpret the information you’re giving them. It’s not easy to understand this illness, but in time, we can figure out how patients want to receive information.” Many noted that leveling power imbalances, by learning some Spanish words or by asking them about things that were meaningful to them, helped to build rapport and establish trust.

#### Patient Empowerment and Activation

Some health care professionals described person-centered care that activates patients to manage their own health as an ideal strategy to improve patient well-being: “You have to take a step back and be careful not to put your values and beliefs on them. Providers struggle because we know what our expectations are, but what are their expectations? We need to sit down and ask them what their own goals are.” Some health care professionals asked patients to take the lead: “Sometimes I’ll say, ‘You’re the manager and I’m the assistant’…so that they are looking at it like they are in charge of their health and how they feel.”

#### Supporting Primary Caregivers

Health care professionals also described that for many Latinx patients, family is the primary source of emotional and decision-making support, and by providing family caregivers with support, the patient received better care: “Sometimes patients say that they are being asked to miss dialysis by family because family wants them to do something else. We’ll do a sit-down family meeting so that they can understand what kidney disease is and why dialysis treatments are important.”

#### Peer Support With Navigation of the Health Care System

Some health care professionals described support from peers as important because peers have the same lived experience with kidney failure and may have faced similar social challenges: “Many of us can’t relate, but someone who comes from their community would have an easier time connecting and could talk to them about kidney transplantation and maybe have more of an impact than the rest of us.” In addition, several health care professionals advocated for having a community health worker who could provide better insight about “what’s going on at home” because you can “really understand so much more about the patient.”

## Discussion

In this qualitative study, we report the perceptions of interdisciplinary health care professionals at inner-city dialysis centers about how social challenges affect the care of Latinx patients with ESKD receiving thrice-weekly hemodialysis. We identified 4 themes conveying barriers to good care: communication issues attributable to low use of language interpreters, lack of culture- and language-concordant educational materials, difficulty with health care access, and psychosocial challenges. Health care professionals also identified facilitators to care that included providing care in ways that are culturally responsive and person centered.

Our finding of communication issues is consistent with previous surveys that found low use of certified language interpreters and reliance on ad hoc interpreters in other health care settings.^21,34,35,36,37,38^ Federal law requires that health care settings provide language access services to patients with LEP and patients who are deaf or hearing impaired, yet access to language services remained a key concern for health care professionals in the dialysis setting. Title VI of the Civil Rights Act of 1964 states that “no person in the US shall, on the ground of race, color, or national origin, be excluded from participation in, be denied the benefits of, or be subjected to discrimination under any program or activity receiving federal financial assistance.”^39^^(p 1)^ The US Supreme Court and the US federal government have treated language as a proxy for national origin: “Because persons of limited English are disproportionately represented in certain national origin groups, the inability to communicate with persons of LEP has the effect of discriminating on the basis of national origin.”^40,41^^(p 82972)^ Violations of federal language access laws are civil rights violations and the cause of approximately 1 of every 40 medical malpractice claims.^42^ Furthermore, numerous studies^36,43,44,45,46,47,48,49,50^ have found that language barriers contribute to health disparities by influencing patient-physician relationships, satisfaction with care, and clinical outcomes.

Use of Spanish-speaking staff or family members as ad hoc language interpreters is discouraged, if not prohibited, and studies^44,51^ have found that ad hoc interpreters increase medical errors. Current US Department of Health and Human Services guidelines allow for ad hoc interpretation only in situations that involve an imminent threat to the safety or welfare of a patient with LEP when no qualified interpreter is immediately available. The use of professional language interpreters over ad hoc interpreters for patients with LEP improves comprehension, clinical outcomes, and patient satisfaction.^52,53,54,55^ To improve care of Latinx with LEP and for legal compliance, dialysis units must develop comprehensive programs that provide and prioritize language access. First, patients should be made aware of their rights for language interpretation. Second, interpreter services should be easily accessible at all points of care via in-person, telephone, or videoconference technologies. Third, the use of ad hoc bilingual staff or family interpreters should be discouraged and occur only in emergency situations. The Centers for Medicare & Medicaid Services could support these efforts by holding dialysis organizations accountable, creating a health equity incentive that enhances reimbursement when language concordant care is provided, and verifying that patient communication needs are met through yearly assessments.

Another key finding included perceptions on strategies to improve care for Latinx patients receiving in-center hemodialysis. One proposed strategy was to provide care that is culturally responsive and personalized to account for and respect the patient’s unique cultural and language needs and perspectives. In the dialysis setting, prior studies^56,57^ indicate that when advice about dietary restrictions does not acknowledge culture, patients have distress in food selection, and language-concordant dietary information improves dietary adherence. The health care professionals who treat Latinx patients in our study reported that their patients experienced social isolation and hopelessness; understanding and working within cultural norms surrounding mental health will be crucial to treating these conditions. Health care professionals also stated that peer support (ie, shared experiences, such as with kidney failure) may be helpful in navigation of the health care system and reduction of social challenges. Community-based approaches using culture- and language-concordant peer-community health care workers who address the social challenges of Latinx patients with kidney failure have been explored as a means to improve patient-centered and clinical outcomes.^16,58,59^ Additional research on the use of culture- and language-concordant community-based interventions is needed.

Some of the challenges that health care professionals reported are issues that face many patients undergoing dialysis, including other minority groups, those living in communities with poor social conditions, and those who lack good kidney care and preparation for ESKD. However, the challenges of language discordance and sensitivity to discordance between mainstream and minority cultures were prominent issues that emerged from health care professionals who treat Latinx patients. Some of these challenges may also inform care for other minority groups. Additional research on the perspectives of other minority groups and other stakeholder groups, such as administrators at dialysis organizations, is needed.

### Limitations

This study has limitations. Although a variety of health care professionals were interviewed, they were from 4 dialysis organizations, and the generalizability of findings to other dialysis centers or organizations is uncertain. In addition, the policies at the dialysis centers varied, and our study took place before the adoption of tablets for language interpretation. Some themes may not have been captured because dialysis practices and protocols vary throughout the country; additional or different challenges may be faced by interdisciplinary health care professionals where practices differ. Social desirability bias may also have caused some participants to censor negative views or attitudes about caring for Latinx individuals.

## Conclusions

Interdisciplinary health care professionals who work at inner-city dialysis centers are at the forefront of providing care to Latinx patients receiving hemodialysis, and these health care professionals identified several challenges to care, including issues with communication and cultural concordance of educational materials, difficulty with health care access, and psychosocial well-being. New policies that require high-quality language interpretation and the availability of culturally concordant educational materials are urgently needed in the dialysis setting. Culturally tailored, community-based interventions that provide support with health care system navigation and social challenges may be a solution and should be tested for improving patient-centered outcomes.

## Notes

Supplement. **eTable.** Interview Guide
