# Supplementary material for: Social and Cultural Challenges in Caring for Latinx Individuals With Kidney Failure in Urban Settings
Source: JAMA Netw Open. 2021 Sep 17;4(9):e2125838. doi: 10.1001/jamanetworkopen.2021.25838 (PMC8449281; doi:10.1001/jamanetworkopen.2021.25838)
Supplement: Supplement. — eTable. Interview Guide [file jamanetwopen-e2125838-s001.pdf]

Supplementary Online Content

Cervantes L, Rizzolo K, Carr AL, et al. Social and cultural challenges in caring for Latinx individuals with kidney failure in urban settings. *JAMA Netw Open*. 2021;4(9):e2125838. doi:10.1001/jamanetworkopen.2021.25838

eTable. Interview Guide

This supplementary material has been provided by the authors to give readers additional information about their work.

© 2021 Cervantes L et al. *JAMA Network Open.*

eTable. Interview Guide

1. What is your role in the care of hemodialysis patients?

2. What issues, if any, exist when providing care for Latinx patients?

3. How do these issues affect your ability to provide care for Latinx patients or for them to care for themselves?

4. Can you describe some of the social challenges experienced by the Latinx patients?

5. What social challenges do you feel are most pressing for your Latinx patients?

6. How, if at all, do these social challenges impact their care?

7. What are your thoughts on how care might be improved for Latinx patients and Latinx patients with limited English proficiency?

© 2021 Cervantes L et al. *JAMA Network Open.*
